# Administration and Value of Antitoxin

**Published:** 1897-09

**Authors:** G. F. Cott

**Affiliations:** Instructor in laryngology University Dispensary; attending laryngologist German Hospital Dispensary; member Erie County Medical Society; New York State Medical Association, etc.; consulting laryngologist Riverside Hospital; physician to City Dispensary; city physician, etc.


### ADMINISTRATION AND VALUE OF ANTITOXIN.

By G. F. COTT, M. D.

Instructor in laryngology University Dispensary; attending laryngologist German Hospital Dispensary; member Erie County Medical Society; New York State Medical Association, etc.; consulting laryngologist Riverside Hospital; physician to City Dispensary; city physician, etc.

AT THE time antitoxin was introduced I was one of the many who accepted its efficacy with much reserve, strictly adhering to the motto, "Be not the first to bid the new drug stay, nor yet the last to cast the old away." I had even gone so far as to translate the article in the *Berliner Klinische Wochenschrift*, by Dr. Hansemann, assistant to Prof. Koch, and read it before a medical club as well as a number of the students at the college dispensary, for the purpose of showing them that there were two sides to the question. My experience at that time was rather limited; since then, however, ample time and opportunity have been afforded for further study of its effects.

There are at present, in the face of overwhelming proof, many physicians who regard the value of antitoxin with suspicion, in some cases due to those rare accidents occasionally recorded, while in others its use was abandoned because all patients did not recover after its administration. The time of its exhibition had also been lost sight of. Records of thousands of cases of diphtheria show that all recover if injected during the first twenty-four hours ; if injected the second day, a small percentage die ; if the third day, a larger percentage. Of course, it is difficult to tell which is the first, second or third day of the disease. A certain rule which I always follow and which has proved correct in something like 100 cases, I have found to be nearly absolute, not one having died in which the prognosis of recovery was given. Later on this will be referred to again.

It does not at all follow that the remedy is valueless because some patients die after being injected. Scepticism will always obtain in medicine, as in religion, and yet it is well to hold fast to that which is good. This paper was prompted by some severe criticisms which recently appeared in a medical journal, for though every physician is entitled to his opinions, facts are facts and are hard to gainsay. A medical journal is for the purpose of spreading knowledge, not of fostering ignorance.

In pharyngeal diphtheria cases often recover without the aid of antitoxin, but occasionally one dies from extension into the larynx before the effect becomes markedly noticeable. Now, accepting the fact (not the theory) that antitoxin has a certain amount of immunising quality, which no rational being with sufficient experience will deny, it is always safe to give a mild dose in all cases of diphtheria, but especially in doubtful ones. By its non-use the physician loads an enormous burden upon his conscience. It is true that too much is expected of antitoxin; its effects are not uniform, except as to final recovery, and then it must have been properly and timely administered. For instance, consider the present condition of the patient, the past history, the record of the family and the like. If the remedy respond well, these considerations may be omitted, but when improvement is slow or at a standstill, it is our duty to become acquainted with the cause. Assuming, of course, that you can place the fullest confidence in the preparation you are injecting, you will appreciate at once how much to use and how often to apply your remedy. All cases do not recover by the use of this agent, but that is no reason why it should be discarded. Chloroform and ether had their bitter enemies and now and then patients die apparently from their effects, still they are being used regardless of the occasional disasters. So also with antitoxin; it has been used long enough for us to weigh the pros and cons, and we find that in France, Germany, England and the United States the death-rate from diphtheria has been reduced from 45 per cent. to 15 per cent.; that even of those cases requiring surgical interference only 27 per cent. died. Of my own cases of intubation and tracheotomy 80 per cent. recovered.

Assuming now that we have reached that stage of evolution where all well-meaning physicians know that antitoxin is the best remedy to be used in diphtheria, whether nasal, pharyngeal or laryngeal (at one time known as membranous croup), let us consider the proper mode of administration. It readily deteriorates when exposed to air, therefore it should be kept tightly corked until ready for use. Wash the skin well (preferably the thigh of a child and arm of an adult), then apply some antiseptic solution with cotton or gauze ; boil both needle and syringe, if that can be done. Draw the entire contents of the vial into the syringe, inject, then place a pledget of sterilised cotton over the puncture as the needle is withdrawn, then bandage.

The most important point to be considered is the amount of antitoxin to be administered at one dose and how often. The dose is never gauged by the number of days of the patient's illness, but always by the physical condition presented. If laryngeal symptoms be at all manifest, a large dose must immediately be exhibited ; if the pharynx only be involved, a dose of moderate size is sufficient. If the nasal cavities are affected, a large dose is required, since there is a large absorbing surface to combat. In the laryngeal type, stenosis and extension of the membrane into the smaller tubes (thus cutting off the supply of oxygen) are generally prevented by a large dose. Antitoxin is but one remedy to be relied upon during the course of the disease and the various symptoms must be treated as they arise. When destructive cell change has taken place antitoxin is practically useless and other remedies may do as well, or better. It is given to prevent this destructive change, but not to stop it. In the laryngeal cases always give the largest and most concentrated dose ; even in very young children, two years or over, to get a rapid effect use the most concentrated article on the market ; repeat it in eight to twelve hours, giving a third or fourth dose if necessary.

All antitoxin preparations in the market are supposed to be of the same standard strength, differing in bulk only, the more bulky the less efficient, or in other words, the slower the effects. The neatest division, I believe, is Mulford's. That house puts out three kinds, differing somewhat in strength of units or normals and in bulk. They have No. 1 standard, 500, 1000, 2000 ; No. 2, potent, and No. 3, extra potent. The most concentrated is No. 3 (extra potent), containing 4 c.c. to 2000 units and should always be given in severe cases. For immunising purposes No. 1 standard is sufficient ; its bulk is only 5 c.c. to 500 units. When a medium dose is required, as in mild cases, No. 2 potent is suitable and very efficient, containing but 4 c.c. to the 1000 units. The other varieties may be used as the physician deems fit. It is well to become acquainted with certain kinds, since they are often given in an emergency when there is no time to look up the subject. I use Mulford's because it has given the best results. The small syringe made by the same firm has proved very efficient. It holds but 5 c. c. and has the additional advantage of allowing the air to be readily and thoroughly expelled. The extra potent may also be administered with an ordinary hypodermic syringe. Those cases of sudden death reported to have followed the injection of antitoxin are supposed to have been caused by injecting air with the liquid, and if such be the case, it is the more necessary to possess a syringe from which the air can be readily expelled.

*Prognosis.*—The pulse is a pretty good indicator as to the effect of the diphtheria poison on the nerve centers and will disclose the presence of fatty degeneration and cell destruction. If at the time of injecting the antitoxin the pulse be strong, even though rapid—160 to 180 with rapid respiration—if intubation had become necessary or tracheotomy been performed, the prognosis is invariably favorable, leaving out of consideration the number of days the patient has been ill, provided the treatment be properly conducted. On the other hand, if the pulse become compressible, indicating a weak heart from toxemia, no amount of antitoxin seems to have much effect. This prognosis does not apply to cases complicated with pneumonia, nephritis, and the like, but to diphtheria only.

After the antitoxin has been administered sustain the heart and fortify the tissues with our old standard remedies, such as corrosive sublimate, strychnine, and the like. Whether or not you wish to omit one or all of the auxiliary drugs, always bear in mind that antitoxin is the sheet-anchor, and if given early, intubation for diphtheritic stenosis will be a thing of the past and tracheotomy will have become obsolete.

Following will be found a record of a few cases, all laryngeal, showing treatment and final results. Diagnosis was confirmed by bacteriological examination.

